# CHRONIC DISABILITIES AND POLYSUBSTANCE USE IN ADULTS AGED 50 + ACROSS 25 US STATES

**DOI:** 10.1093/geroni/igad104.3224

**Published:** 2023-12-21

**Authors:** John Massey, Mika Thompson, Laura Johnston, Michael Phillips

**Affiliations:** University of Hawaii at Manoa, Honolulu, Hawaii, United States; University of Hawaii at Manoa, Honolulu, Hawaii, United States; University of Hawaii at Manoa, Honolulu, Hawaii, United States; University of Hawaii at Manoa, Honolulu, Hawaii, United States

## Abstract

Given an aging demographic that reports higher substance use patterns than preceding generations, as well as variability in cannabis legality at the state level, substance use and abuse is an area of concern for older adults experiencing chronic disabilities. Examining trends for polysubstance use for three of the most used substances (nicotine, alcohol, and cannabis) allows for possible insight into use patterns exhibited by older adults, particularly those with disabilities. Using national data across 25 states from 2020-2021 BRFSS, we investigated recent substance use for adults aged 50 and over (N=200,793). We conducted multinomial regression analyses to examine polysubstance use by individual disabilities (including hearing, vision, cognition, mobility, self-care, and independent living) and any disability. Our results indicated that 36.72% reported at least one disability, while 1.46% reported recent polysubstance use of alcohol, nicotine, and cannabis. All but one (mobility) of the individual disabilities were associated with higher odds of polysubstance use. Having any disability was associated with 42% higher odds of polysubstance use (OR: 1.42, 95%CI: 1.30-1.56) compared to no use. Understanding trends associated with polysubstance use for the three most commonly used substances in the U.S. for older adults 50+ with disabilities may influence future policy discussions, public health campaigns, efforts to educate older adults on the potential harms associated with polysubstance use, and at improving health equity and population health outcomes.

